# Interpreting ^123^I–ioflupane dopamine transporter scans using hybrid scores

**DOI:** 10.1186/s41824-018-0028-0

**Published:** 2018-05-21

**Authors:** Kenneth J. Nichols, Brandon Chen, Maria B. Tomas, Christopher J. Palestro

**Affiliations:** 1 Department of Radiology, Donald and Barbara Zucker School of Medicine at Hofstra/Northwell, Hempstead, NY USA; 2 Division of Nuclear Medicine and Molecular Imaging, Northwell Health, 270-05 76th Avenue, New Hyde Park, NY 11040 USA

**Keywords:** ^123^I–ioflupane, Dementia with Lewy bodies, Parkinson’s disease, Dopamine transporters, DaT, Brain SPECT

## Abstract

**Background:**

Dopamine transporter (DaT) ^123^I–FP-CIT scans most commonly are interpreted visually. Alternatively, absolute quantitation of radiopharmaceutical uptake may improve scan accuracy. However, neither approach accomodates dependence of striatal uptake on age and gender. We investigated whether demographic indexing of visual and numerical variables improve discrimination of patients with essential tremor (ET), Parkinson’s disease (PD), and dementia with Lewy bodies (DLB).

**Methods:**

Data of 132 consecutive patients undergoing DaT SPECT scans were reviewed retrospectively. The clinical impression in the latest neurology note was utilized as the final clinical diagnosis. Caudate and putamen specific binding ratio (PSBR) were computed. ^123^I calibration phantoms were constructed to enable absolute quantitation of putamen radiopharmaceutical uptake. A single experienced nuclear medicine physician graded visual certainty on a 3-level scale. Demographic indexing normalized metrics to published normal PSBR values. Methods were compared by simultaneous ROC analyses to identify the technique of maximal accuracy.

**Results:**

Thirty-four patients (26%) were diagnosed with ET, 85 (64%) with PD, 6 (5%) with multiple system atrophy, and 7 (5%) with DLB. For discriminating DLB from PD, visual analysis was significantly less specific and accurate than the other techniques. However, indexing significantly improved specificity and accuracy of visual scores, such that indexed visual scores were statistically equivalent to all other methods. Indexed PSBR yielded essentially the same results as non-indexed PSBR, for which highest overall test efficacy was achieved.

**Conclusions:**

Our results in this small series of patients with DLB suggest that if ^123^I–FP-CIT visual scores are to be used to discriminate DLB from other neurologic disorders, demographic indexing should be applied. However, best results overall are obtained using quantified parameters, regardless of whether or not demographic indexing is applied to these values.

## Background

Dopamine transporter (DaT) ^123^I–ioflupane scans are performed to help discriminate patients with essential tremor (ET), for which radiopharmaceutical uptake is intense and symmetric in caudate and putamen structures, from patients with Parkinson’s syndrome (PS). DaT scans utilize the radioligand [^123^I] N-x-flouropropyl-2b-carbomethoxy-3b-(4-iodophenyl) nortropane (FP-CIT) and single photon emission computed tomography (SPECT) to detect dopaminergic dysfunction in the brain. FP-CIT binds to DaT, a presynaptic protein in nigrostriatal neurons responsible for dopamine reuptake from the synapatic cleft. DaT are found in high concentrations in the striatum (caudate and putamen). Loss of nigrostriatal neurons results in decreased dopamine concentrations in the striatum, and DaT levels correlate with dopamine concentrations in the striatum (Kish et al., 1988; Andringa et al., 2005). Nigrostriatal neuronal dysfunction or loss of substantia nigra neuronal cell bodies results in reduced binding of FP-CIT (Walker & Walker, 2009). In Parkinson’s disease, loss of dopaminergic activity is progressive and first occurs in the contralateral putamen (Marshall & Grosset, 2003).

**Fig. 1:**
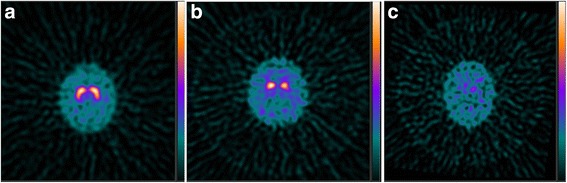
DaT scan examples. DaT scan transaxial SPECT sections for: (**a**) a patient with essential tremor, showing high symmetric uptake in both caudate and putamen regions; (**b**) a patient with Parkinson’s disease, showing markedly reduced putamen activity and asymmetric caudate activity; (**c**) a patient with dementia with Lewy bodies, showing nearly absent caudate and putamen uptake

Parkinson’s disease (PD), however, is not the only cause of PS; other causes include progressive supranuclear palsy, multiple system atrophy, corticobasal degeneration, and dementia with Lewy bodies (DLB) (Fig. 1) (Marshall & Grosset, 2003). DLB is the second most common form of neurodegenerative dementia, after Alzheimer’s disease, accounting for 4–8% of all newly diagnosed dementia cases (Vann Jones & O’Brien, 2014; van der Zande et al., 2016). DLB is caused by abnormal deposition of alpha-synuclein protein in dopaminergic nigrostriatal neurons as well in other portions of the brain. The core clinical features of DLB include fluctuating cognition, visual hallucinations, and movement symptoms. As such, the clinical presentation of DLB overlaps with Parkinson’s disease as well as with Alzheimer’s disease, compromising the ability to form a definitive diagnosis (van der Zande et al., 2016). While patients with DLB and Alzheimer’s have similar symptoms in early disease stages, the course of these diseases and their management is substantially different for patients with DLB from those with Alzheimer’s (Bostrom et al., 2007a; Bostrom et al., 2007b).

The main use of DaT scanning is in patients with a movement disorder to help differentiate ET from tremor due to neurodegenerative parkinsonism, but DaT scans also are used to help differentiate patients with DLB from those with Alzheimer’s (Walker et al., 2015). Whereas an abnormal DaT scan has been included as a suggestive diagnostic feature for DLB (McKeith et al., 2005), a normal DaT scan is consistent with Alzheimer’s disease, ET, drug-induced parkinsonism, or in some cases vascular parkinsonism (Gerschlager et al., 2002; Sixel-Döring et al., 2011; Djang et al., 2012; Utiumi et al., 2012).

DaT SPECT scans most commonly are interpreted visually, and scoring systems have been proposed to aid in qualitative evaluation (Benamer et al., 2000). Absolute quantitation of radiopharmaceutical uptake has the potential to improve accuracy of nuclear tomography (Gnesin et al., 2016). However, neither use of visual scores nor absolute quantitation takes into account observed dependence of caudate and putamen uptake on age and gender (Nobili et al., 2013). Therefore, we conducted this investigation to determine whether demographic indexing, i.e., adjusting visual scores and quantitative uptake measurements for previously documented DaT SPECT scan differences associated with age and gender, can improve discrimination of patients with PD from those with DLB.

## Methods

### Compliance with ethical standards

The Institutional Review Board approved this retrospective study and the requirement to obtain informed consent was waived (IRB #: 13-139B). All data were handled in compliance with the Health Insurance Portability and Accountability Act of 1996. None of the authors have any conflicts of interest to disclose in relation to this investigation.

### Patients

From August 2011 through July 2016, 280 consecutive patients who underwent DaT scanning at our institution were reviewed retrospectively. The clinical indication for all examinations was movement disorder. Clinical follow-up was obtained primarily through institutional ambulatory electronic health records. Follow-up was successfully obtained for 132 patients (68 ± 11 years; 76 males; 56 females). For each patient, all neurology clinic notes prior to and after the DaT scan were reviewed. The clinical impression in the latest neurology note was utilized as the presumptive final clinical diagnosis. For each patient, age and sex were tabulated at the time of the DaT scan. The amount of injected activity for each patient was obtained from the Division of Nuclear Medicine and Molecular Imaging patient charts, to enable quantitation of absolute uptake.

### Data acquisition

Patients were pretreated with Lugol's solution approximately one hour before radiopharmaceutical administration. SPECT imaging was performed 3–5 h after IV injection of 185 MBq (5 mCi) of ^123^I–FP-CIT, according to standardized guidelines (Darcourt et al., 2010). Data were acquired according to the manufacturer’s suggested protocol, employing a 15% energy window centered at 159 keV, 128 × 128 matrixes, zoom factor of 1.0, pixel size of 4.66 mm, at 30 s/projection for 128 projections, for a total acquisition time of 34–35 min, using low energy high resolution collimators. The same dual detector rotating gamma camera system (Skylight, Philips Inc.) was used for all patient acquisitions. All tomograms were reconstructed by filtered backprojection (with Butterworth cutoff = 0.5 and order = 8) as per the manufacturer, and corrected for attenuation by the Chang method (linear attenuation coefficient μ = 0.11).

**Fig. 2:**
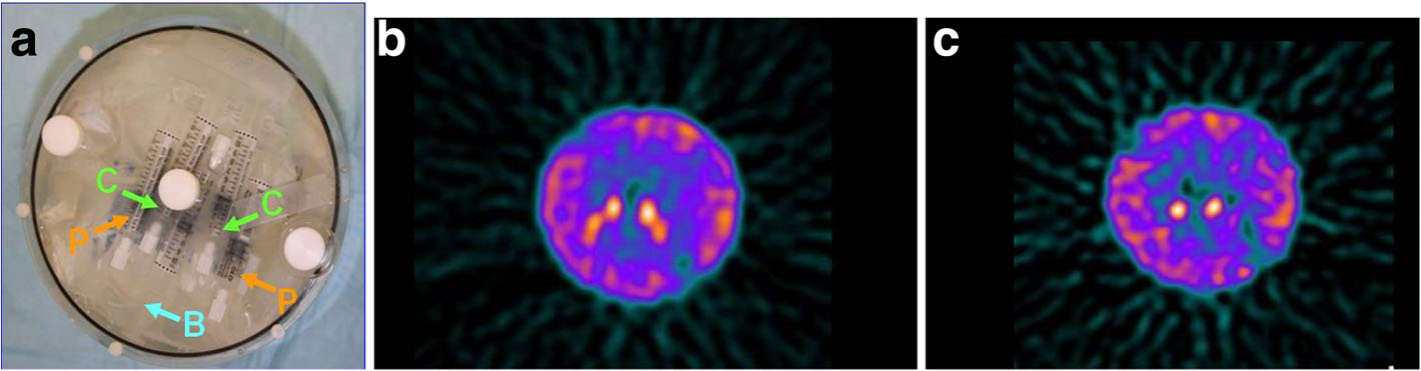
Physical phantom. **a** Physical phantom with syringes loaded with radioactivity representing caudate (C) and putamen (P) structures, and radioactivity loaded into surrounding water background (B). Reconstructed phantom images for simulations representing (**b**) essential tremor and (**c**) Parkinson’s disease

To enable absolute quantitation of radiopharmaceutical uptake, phantoms were constructed using 1.7–7.0 MBq (20–80 μCi) ^123^I in saline in four 5-mL syringes to simulate caudate nuclei and putamina in a 6000 mL water phantom with ^123^I background activity (Fig. 2a). Each syringe was filled with activity for 1 mL of its 5 mL volume to simulate left and right caudate nuclei and putamina. The same gamma camera system used for all patient data acquisitions was used for all phantom acquisitions. Seven simulations were performed to replicate typical image data acquired for patients with ET, using identical data acquisition and tomographic reconstruction procedures as was performed for clinical data, with the only exception being that times per projection were adjusted to provide simulated striatal and background counts typical of mean clinical counts (Fig. 2b and c).

### Quantitative parameters

To obtain striatal counts, a medical physicist manually drew regions of interest on all clinical scans and phantom simulations to determine caudate, putamen and background counts. The relative measurement of caudate-to-background specific binding ratio (CSBR) was computed as:

1 $$ \mathrm{CSBR}=\left(\mathrm{C}-\mathrm{B}\right)/\mathrm{B} $$

where B is background counts per pixel and C is the minimum of left or right caudate mean count per pixel. The relative measurement of putamen-to-background specific binding ratio (PSBR) was computed similarly as:

2 $$ \mathrm{PSBR}=\left(\mathrm{P}-\mathrm{B}\right)/\mathrm{B} $$

where P is the minimum of left or right putamen mean counts per pixel.

Reconstructed phantom data were used to calibrate caudate absolute quantitation (CAQ) and putamen absolute quantitation (PAQ). As with CSBR and PSBR ratios defined above, the minimum of left or right mean striatal counts were used for caudal and putaminal counts. Both CAQ and PAQ were corrected for mean background counts. Because there was a range of injected activities for clinical studies, the physical unit chosen for absolute quantitation was the percent uptake of injected activity per mL of brain tissue. Patient caudate nuclei and putamina each were assumed to have a volume of 1 mL. The ratio of counts per pixel in the caudate nuclei and putamina for clinical studies versus phantom studies then yielded estimates of absolute quantitation of percent uptake of injected activity per mL.

### Demographic indexing

To apply demographic indexing, all metrics were normalized to CSBR and PSBR values that have been published for normal volunteers (Nobili et al., 2013). This was accomplished using published expected values of striatal-to-background count ratios:

3 $$ \mathrm{Normal}\ \mathrm{CSBR}=6.8\hbox{--} 0.02{73}^{\ast}\mathrm{age}\ \mathrm{for}\ \mathrm{males} $$

4 $$ \mathrm{Normal}\ \mathrm{CSBR}=7.232\hbox{--} 0.02{73}^{\ast}\mathrm{age}\ \mathrm{for}\ \mathrm{females} $$

5 $$ \mathrm{Normal}\ \mathrm{PSBR}=6.702\hbox{--} 0.03{39}^{\ast}\mathrm{age}\ \mathrm{for}\ \mathrm{males} $$

6 $$ \mathrm{Normal}\ \mathrm{PSBR}=7.116\hbox{--} 0.03{39}^{\ast}\mathrm{age}\ \mathrm{for}\ \mathrm{females} $$

Each CSBR and PSBR value was divided by the expected value for a normal subject of the age and gender of the patient. In addition to indexing ratios of striatal counts to background counts, we also examined indexed CAQ, indexed PAQ, and indexed visual scores using these same normalizing factors (Eqs. 3–6), which required inherently assuming that background counts are not dependent on age.

### Visual readings

A single nuclear medicine physician, with more than 20 years experience in the field and more than 7 years experience in reading (superscript)123-I ioflupane studies, graded all clinical studies, blinded to previous reports, final diagnoses, and quantified parameters and studies. The assigned grades on a 3-point scale were: 0 = “definitely normal,” 1 = “equivocal,” and 2 = “definitely abnormal.” The same physician also dichotomously graded each scan as visually appearing to have symmetric or asymmetric striatal uptake.

To form an hybrid score combining visual scores with information as to gender and age dependence of ^123^I–FP-CIT, we divided the visual score by the normal PSBR value for males or females (Eqs. 5 and 6), in order to obtain the indexed visual score.

### Statistics

All values are reported as means with 1 standard deviation. Analysis of variance (ANOVA) tested multiple variables simultaneously to detect differences among disease categories. The normality of distributions of continuous variables was assessed by the chi-square test. The t-test was used to detect differences between means of normal distributions; otherwise, the Wilcoxon test was used. Chi-square analysis of proportions was used to determine if ratios were significantly different. Power analyses were used to determine if differences were significant for patient sub-groups with small sample sizes. Linear regression was used to test whether background counts were correlated with age. ROC analyses were performed to analyze the ability of different metrics to discriminate groups of patients by disease states. ROC analyses provided discrimination thresholds to dichotomize variables, from which sensitivity, specificity and accuracy were determined. All analyses were performed using commercially available software (MedCalc Statistical Software version 18 (MedCalc Software bvba, Ostend, Belgium; http://www.medcalc.org; 2018)).

## Results

Mean follow up time from DaT scans to final diagnoses was 1.3 ± 1.1 yrs., at which time 34 (26%) had ET, 85 (64%) had PD, 6 (5%) had multiple system atrophy, and 7 patients (5%) had DLB.

Background counts were uncorrelated with age, both for all patients (r = − 0.08, *p* = 0.33) and for the subgroup of patients with ET (r = − 0.09, *p* = 0.58). ANOVA showed no differences in background counts among disease groups (F-ratio = 1.1, *p* = 0.37), nor were background counts different between male and female patients with ET (108 ± 23 versus 110 ± 27 counts/pixel, *p* = 0.38), so that the assumption of independence of background counts with age and sex were considered to be valid for subsequent analyses.

### Discriminating ET from other disease states

**Fig. 3:**
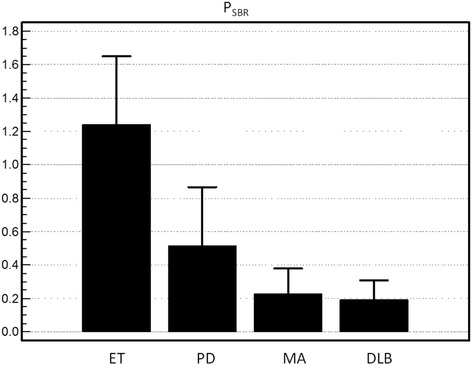
Putamen specific binding ratios. Putamen specific binding ratio (P_SBR_) plotted for patients with essential tremor (ET), Parkinson’s disease (PD), multiple system atrophy (MA) and dementia with Lewy bodies (DLB)

ANOVA demonstrated that visual scores, CSBR, PSBR, CAQ and PAQ all were significantly different among disease states (*p* <  0.001), with F-ratios > 12.1. The highest F-ratio (41.2) was found for indexed PSBR, indicating this metric had the greatest inter-group variability (Table 1, Fig. 3). All metrics were significantly different between ET and all other disease states. Patients with ET had mean age of 70 ± 11 yrs. (range: 49–88 years), for whom CSBR was 2.08 ± 0.52 and PSBR was 1.24 ± 0.42. Several metrics were different between patients with DLB and those with PD, but none were different between patients with MA and DLB (Table 1).

**Table 1:** Visual and quantitative parameters for disease states

	ET (*N* = 34)	PD (*N* = 85)	MA (*N* = 6)	DLB (*N* = 7)	ANOVA F-ratio
Visual	0.81 ± 0.86	1.77 ± 0.55*	2.00 ± 0.00*	1.86 ± 0.38*	18.9
C_SBR_	2.08 ± 0.52	1.30 ± 0.54*	0.53 ± 0.23*†	0.80 ± 0.25*†	28.5
P_SBR_	1.24 ± 0.42	0.51 ± 0.36*	0.22 ± 0.17*†	0.28 ± 0.18*†	38.9
CAQ	5.34 ± 2.04	3.8 ± 1.76*	1.81 ± 1.39*†	1.94 ± 1.12*†	12.1
PAQ	2.11 ± 1.06	0.94 ± 0.61*	0.59 ± 0.49*	0.45 ± 0.40*†	23.8
Indexed Visual	0.18 ± 0.19	0.39 ± 0.13*	0.44 ± 0.02*	0.44 ± 0.09*†	17.2
Indexed C_SBR_	0.42 ± 0.11	0.25 ± 0.11*	0.10 ± 0.04*	0.16 ± 0.05*	29.7
Indexed P_SBR_	0.28 ± 0.09	0.11 ± 0.08*	0.05 ± 0.04*	0.07 ± 0.05*	41.2
Indexed CAQ	5.31 ± 1.09	3.67 ± 1.67*	1.74 ± 1.34*†	1.98 ± 1.11*†	13.2
Indexed PAQ	2.38 ± 1.27	1.02 ± 1.27*	0.64 ± 1.27*	0.53 ± 0.48*†	24.3

*ET* essential tremor, *PD* Parkinson’s disease, *MA* multiple system atrophy, *DLB* dementia with Lewy bodies, *ANOVA* analysis of variance, *C*_*SBR*_ caudate specific binding ratio, *P*_*SBR*_ putamen specific binding ratio, *CAQ* minimum caudate absolute quantitative uptake, *PAQ* minimum putamen absolute quantitative uptake

* *p* < 0.05 versus ET; † *p* < 0.05 versus PD

For discriminating patients with ET from patients with PD, or for discriminating patients with ET from patients with all other disease states, ROC analyses indicated that PSBR had the highest accuracy of 86–87% (Tables 2 and 3). Specificity was significantly lower for visual readings, CAQ, PAQ and indexed visual readings compared to 89% specificity of PSBR (Tables 2 and 3).

**Table 2:** ROC result for discriminating patients with ET from those with PD

	Threshold for discrimination	Sensitivity	Specificity	Dichotomous Accuracy
Visual	> 1	82%	72%*	79%
C_SBR_	< 1.56	79%	86%	81%
P_SBR_	< 0.76	85%	89%	86%
CAQ	< 4.73	78%	56%*	71%*
PAQ	<=1.70	87%	67%*	81%
Indexed Visual	> 0.24	85%	72%*	81%
Indexed C_SBR_	< 0.31	77%	86%	79%
Indexed P_SBR_	<=0.15	79%	92%	83%
Indexed CAQ	<=4.96	54%*	81%	62%*
Indexed PAQ	<=1.65	82%	75%	81%

*p < 0.05 versus P_SBR_

**Table 3:** ROC result for discriminating patients with ET from those with all other disease states (i.e., PD, MA and DLB)

	Threshold for discrimination	Sensitivity	Specificity	Dichotomous Accuracy
Visual	> 1	84%	71%*	80%
C_SBR_	< 1.56	82%	85%	83%
P_SBR_	< 0.76	87%	88%	87%
CAQ	< 5.12	82%	53%*	74%*
PAQ	<=1.70	89%	68%*	83%
Indexed Visual	> 0.24	87%	71%*	83%
Indexed C_SBR_	< 0.31	80%	85%	81%
Indexed P_SBR_	<=0.16	82%	91%	84%
Indexed CAQ	<=3.65	59%*	79%	64%*
Indexed PAQ	<=1.66	85%	74%	82%

*p < 0.05 versus P_SBR_

### Discriminating patients with DLB

Indexed CAQ was the metric with the highest dichotomous accuracy to discriminate patients with DLB from those with PD (Table 4). All metrics had similar sensitivity (57%–100%). With only 7 cases of patients with DLB, power analysis indicated there were insufficient cases to detect significant differences among metrics for sensitivity, but also indicated that differences for specificity and accuracy were statistically significant. Visual readings, CSBR and indexed CSBR measurements had significantly lower specificity than the 92% specificity of Indexed CAQ (Table 4). The improvement in specificity of visual scores by demographic indexing was significant (6% versus 86%, *p* <  0.0001).

**Table 4:** ROC result for discriminating patients with DLB from those with PD

	Threshold for discrimination	Sensitivity	Specificity	Dichotomous Accuracy
Visual	> 1	100%	6%*	13%*
C_SBR_	<=0.87	100%	60%*	63%*
P_SBR_	<=0.15	57%	87%	85%
CAQ	<=1.71	71%	87%	86%
PAQ	<=0.32	71%	86%	85%
Indexed Visual	> 0.46	57%	86%	84%
Indexed C_SBR_	<=0.19	85%	66%*	67%*
Indexed P_SBR_	<=0.039	57%	82%	80%
Indexed CAQ	<=1.67	71%	88%	87%
Indexed PAQ	<=0.41	71%	84%	83%

*p < 0.05 versus Indexed CAQ

**Fig. 4:**
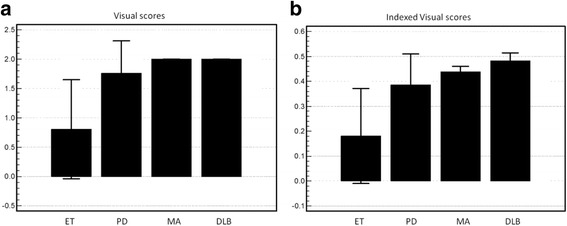
Visual scores. **a** Visual scores plotted for patients with ET, PD, MA and DLB. 4 (**b**) Indexed visual scores plotted for patients with ET, PD, MA and DLB

Similarly, Indexed CAQ was the most accurate means of discriminating patients with DLB from patients with all other disease states (Table 5). Specificity of visual readings, CSBR and indexed CSBR measurements were significantly lower than the 90% specificity of Indexed CAQ (Table 5), but indexed visual scores had significantly higher specificity than non-indexed visual scores (88% versus 31%, *p* <  0.0001) (Table 5, Fig. 4).

**Table 5:** ROC result for discriminating patients with DLB from all other patients (i.e., ET, PD and MA)

	Threshold for discrimination	Sensitivity	Specificity	Dichotomous Accuracy
Visual	> 1	86%	31%*	34%*
C_SBR_	<=0.87	100%	67%*	69%*
P_SBR_	<=0.15	86%	65%*	66%*
CAQ	<=1.71	71%	89%	88%
PAQ	<=0.32	71%	88%	87%
Indexed Visual	> 0.47	57%	88%	86%
Indexed C_SBR_	<=0.19	86%	72%*	73%*
Indexed P_SBR_	<=0.039	86%	60%*	61%*
Indexed CAQ	<=1.67	71%	90%	89%
Indexed PAQ	<=0.41	71%	86%	86%

*p < 0.05 versus Indexed CAQ

## Discussion

Neither demographic indexing nor use of absolute quantitation improved ability to discriminate patients with DLB compared to conventional putamen specific binding ratios. Nonetheless, we found that while visual scores were not as specific or as accurate as quantitative measures for discriminating patients with DLB from other neurologic diseases, demographic indexing to produce a hybrid visual score did significantly improve test specificity, to the point that indexed visual scores were statistically equivalent to quantified metrics for this purpose. As shown in Fig. 4, demographic indexing effectively stretched and adjusted the range of visual measurements, producing more distinct differences among disease states, as compared to non-indexed visual scores.

In our study, the most common indication for DaT scan was to discriminate essential tremor from neurodegenerative Parkinsonism. Diagnostic uncertainty is not uncommon with clinical evaluation. The literature indicates that DaT scans are useful, particularly in patients who present with unclear or atypical symptoms. In a 3-year prospective study, initial clinical diagnosis of Parkinson’s disease was found to have a specificity of 46% and sensitivity of 93% when compared to diagnosis after 3 year clinical follow-up, compared to 97% and 78% with an initial DaT scan (Cummings et al., 2011). In another study, DaT scan was shown to have sensitivity for parkinsonism of up to 97% and a specificity for essential tremor up to 100% (Benamer et al., 2000). A normal DaT scan can essentially exclude a diagnosis of neurodegenerative PS. One study found that 97% of patients with a normal DaT scan do not have a form of neurodegenerative PS at 3 year follow-up (Marshal et al., 2009). A DaT scan often changes management, and medication or diagnosis changes were seen in 59% of patients following a DaT scan at a major tertiary care center (Bega et al., 2015).

On the other hand, more than 10% of patients diagnosed with Parkinson’s disease based on clinical symptoms have normal DaT scans, among whom about 30% have progressively worsening measureable cognitive impairment 2 years after imaging (Wyman-Chick et al., 2016). It can be challenging to visually evaluate DaT scans for patients with ET, considering that DaT scans appear the same for normal volunteers as for patients with ET; nonetheless, striatal-to-background specific binding ratios are measurably lower for patients with ET than for normal subjects (Gerasimou et al., 2012).

Our patients with ET, aged 70 ± 11 yrs. (range: 49–88 yrs), had CSBR of 2.08 ± 0.52 and PSBR of 1.24 ± 0.42, values that were lower (*p* <  0.001) than mean published values of 2.5 ± 0.3 and 1.9 ± 0.2, respectively, of patients with ET aged 64 ± 11 yrs. (range 28–81 yrs) (Gerasimou et al., 2012). In part, this reflects the fact that we chose to use minimum counts rather than means; our mean caudate SBR’s were 2.3 ± 0.5 and putamen SBR’s were 1.5 ± 0.4.We chose to use minimum rather than mean counts as these will be more abnormally low for patients with neurologic disorders. Other investigators have reported still higher mean values (Yokoyama et al., 2017), with mean CSBR and anterior PSBR values of 2.8 and 2.3 of a quantitative software package, “DaTQUANT,” for non-Parkinson’s disease patients aged 26–83. Various methodology differences will likely produce different mean values for ^123^I–FP-CIT DaT scan striatal counts at different institutions. While taking ratios of counts rather than absolute counts should help to provide some commonality, and use of similar post-injection interval and collimator types also would help, there are unavoidable differences regarding data processing. Different manufacturers implement their reconstruction algorithms with different approaches, assumptions, approximations and scaling factors. The reason we chose filtered backprojection with a Butterworth filter is that the US manufacturer of ^123^I–FP-CIT continues to recommend this approach, even though reconstruction methods that incorporate collimator resolution information, such as OSEM, likely produce more reliable striatal count recovery. Comparing results of tomograms processed by FBP to those processed by OSEM could prove worthwhile, but was beyond the scope of our investigation.

We did not include patient weight and height in our indexing, as some studies have shown that DaT availability is unrelated to body mass index over a wide range of values among healthy subjects (Thomsen et al., 2013).

Our retrospective study correlated initial DaT scan results with a reference standard clinical diagnosis at an average of 1.3 years later. We demonstrated utility of DaT scans in excluding the diagnosis of a neurodegenerative PS. This is important, as Parkinson’s disease is often initially overdiagnosed, and unnecessary treatment and patient concern can be avoided (Marshal et al., 2009; Jennings et al., 2004). In our study, visual analysis of DaT scans for discriminating between essential tremor and neurodegenerative PS had 84% sensitivity (Table 3). Sensitivity of visual readings by blinded observers of 95% has been reported in a multicenter study that included patients with established diagnoses of Parkinson’s disease and essential tremor (Benamer et al., 2000). However, that study reported a specificity of 93%, while our study resulted in only 72% specificity. This may be due in part to the fact that a consensus visual assessment was made between four nuclear medicine physicians and a neurologist in that study, while in our study a single experienced nuclear medicine physician evaluated DaT scans, which is the way these studies typically are interpreted. Another multicenter study, which involved follow-up of patients with bradykinesia and also rigidity, tremor, or postural instability and patients with Findley and Koller criteria for definite or probable essential tremor, reported sensitivity of 78% and specificity of 97% for baseline DaT scans (Cummings et al., 2011). While our specificity with regard to visual diagnosis is lower than what is reported in the literature, this may be due partly to lack of inclusionary criteria for patients in our study. Our specificity may in part reflect the complex and atypical patient presentations encountered in clinical practice.

Our study also involved quantitative analysis of putamen and caudate counts with adjustment for background counts. Quantitative parameters are likely affected by patterns of progressive dopamine loss in Parkinson’s disease, in which the contralateral putamen is first affected (Marshall & Grosset, 2003; Fearnley & Lees, 1991). This is consistent with a study involving essential tremor and Parkinson’s disease patients with sequential DaT scans (Isaias et al., 2010), in which quantitative analysis revealed overlap between essential tremor and Parkinson’s disease for caudate uptake, as well as an annual rate of decline of 7.3% for uptake in the contralateral putamen. Additionally, this suggests that quantitative analysis of the putamen can aid in early diagnosis of Parkinson’s disease, and therefore allow earlier initiation of treatment, perhaps prior to onset of clinical symptoms.

We found that PSBR ratio < 0.76 was more specific (89%) than visual analysis (72%) in discriminating between Parkinson’s disease and essential tremor (Table 2). The trend of increasing specificity with quantitative analysis is consistent with a study comparing initial DaT scan imaging diagnoses with 6-month clinical follow-up (Jennings et al., 2004), which reported an increase in specificity from 80% to 100% when utilizing a cutoff of 30% decrease in age-corrected putamen uptake from normal controls to define abnormal DaT scans. Quantitative analyses may be important in decreasing the false positive rate of DaT scans. Our study found that quantification of putamen activity with DaT scans was accurate in discriminating patients with Lewy body dementia from Parkinson’s disease. This is consistent with a prior study demonstrating a differential pattern of striatal dopaminergic loss between Lewy body dementia and Parkinson’s disease. Walker, et al., found a significant difference in caudate binding, with DLB patients demonstrating lower binding than Parkinson’s disease patients (Walker et al., 2004). They also found a predilection for posterior putamen involvement in Parkinson’s disease when compared to DLB, and that caudate to putamen uptake ratio was significantly different in Parkinson’s disease patients than in DLB patients.

### Limitations

The low numbers of Lewy body dementia patients limits our study. Although clinical follow-up was obtained through neurology notes, our study did not involve a standardized clinical assessment for all patients included in the study. Also, as with many prior studies with DaT scans, our study used clinical follow-up as the diagnostic reference standard, which intrinsically cannot prove greater accuracy than thorough clinical evaluation itself. In addition, the caudate and putamen counts in our study were obtained through manually drawn of regions of interest. Use of standardized segmentation maps would have reduced uncertainty in the size and shapes of these regions (Nobili et al., 2013).

## Conclusions

Our results in this small series of patients with dementia with Lewy bodies suggest that if ^123^I–FP-CIT visual scores are to be used to discriminate DLB from other neurologic disorders, demographic indexing should be applied. However, best results overall are obtained using quantified parameters, regardless of whether demographic indexing is applied to these values.
